# Poly[μ_2_-aqua-μ_2_-(pyrazine-2-carboxyl­ato)-lithium]

**DOI:** 10.1107/S1600536812024683

**Published:** 2012-06-16

**Authors:** Wojciech Starosta, Janusz Leciejewicz

**Affiliations:** a Institute of Nuclear Chemistry and Technology, ul.Dorodna 16, 03-195 Warszawa, Poland

## Abstract

The structure of the title compound, [Li(C_5_H_3_N_2_O_2_)(H_2_O)]_*n*_, contains an Li^I^ ion with a distorted trigonal–bipyramidal coordination environment involving the N and O atoms of pyrazine-2-carboxyl­ate ligands with a bridging carboxyl­ate group, and two aqua O atoms also in a bridging mode. The symmetry-related Li^I^ ions bridged by a carboxyl­ate O atom and a coordinating water O atom form an Li_2_O_2_ unit with an Li⋯Li distance of 3.052 (4) Å, which generates mol­ecular ribbons propagating in the *c*-axis direction. The ribbons are held together by a network of O—H⋯O hydrogen bonds in which the coordinating water mol­ecules act as donors and the carboxyl­ate O atoms as acceptors.

## Related literature

For the crystal structure of an Li^I^ complex with a 3-amino­pyrazine-2-carboxyl­ate ligand, see: Starosta & Leciejewicz, (2010 ▶) and for the crystal structure of an Li^I^ complex with a 5-methyl­pyrazine-2-carboxyl­ate ligand, see: Starosta & Lecieje­wicz, (2011*a* ▶). The structures of complexes with pyrid­azine-3-carboxyl­ate and pyridazine-4-carboxyl­ate ligands were reported by Starosta & Leciejewicz, (2011**b* ▶,c* ▶). The structure of a complex with a pyrimidine-2-carboxyl­ate ligand was also determined (Starosta & Leciejewicz, 2011*d* ▶).
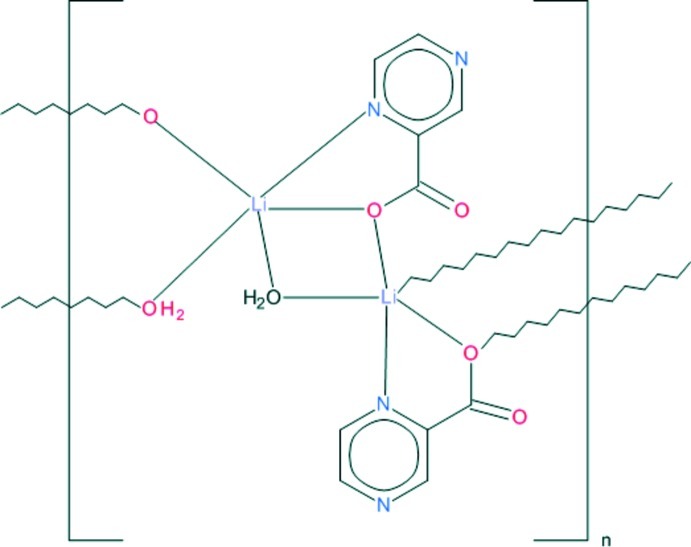


## Experimental

### 

#### Crystal data

[Li(C_5_H_3_N_2_O_2_)(H_2_O)]
*M* *_r_* = 148.05
Orthorhombic, 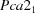

*a* = 24.433 (5) Å
*b* = 4.7861 (10) Å
*c* = 5.6385 (11) Å
*V* = 659.4 (2) Å^3^
*Z* = 4
Mo *K*α radiation
μ = 0.12 mm^−1^
*T* = 293 K
0.35 × 0.18 × 0.13 mm

#### Data collection

Kuma KM-4 four-cricle diffractometer
Absorption correction: analytical (*CrysAlis RED*; Oxford Diffraction, 2008 ▶) *T*_min_ = 0.972, *T*_max_ = 0.995
1586 measured reflections
1056 independent reflections
813 reflections with *I* > 2σ(*I*)
*R*_int_ = 0.078
3 standard reflections every 200 reflections intensity decay: 4.4%

#### Refinement

*R*[*F* ^2^ > 2σ(*F* ^2^)] = 0.041
*wR*(*F* ^2^) = 0.116
*S* = 1.09
1056 reflections
108 parameters
1 restraint
H atoms treated by a mixture of independent and constrained refinement
Δρ_max_ = 0.31 e Å^−3^
Δρ_min_ = −0.30 e Å^−3^

### 

Data collection: *KM-4 Software* (Kuma, 1996 ▶); cell refinement: *KM-4 Software*; data reduction: *DATAPROC* (Kuma, 2001 ▶); program(s) used to solve structure: *SHELXS97* (Sheldrick, 2008 ▶); program(s) used to refine structure: *SHELXL97* (Sheldrick, 2008 ▶); molecular graphics: *SHELXTL* (Sheldrick, 2008 ▶); software used to prepare material for publication: *SHELXTL*.

## Supplementary Material

Crystal structure: contains datablock(s) I, global. DOI: 10.1107/S1600536812024683/kp2421sup1.cif

Structure factors: contains datablock(s) I. DOI: 10.1107/S1600536812024683/kp2421Isup2.hkl

Supplementary material file. DOI: 10.1107/S1600536812024683/kp2421Isup3.cml

Additional supplementary materials:  crystallographic information; 3D view; checkCIF report

## Figures and Tables

**Table 1:** Selected bond lengths (Å)

Li1—O1	2.080 (6)
Li1—N1	2.190 (6)
Li1—O3	2.013 (6)
Li1—O3^i^	2.032 (5)
Li1—O1^i^	2.237 (6)

Symmetry code: (i) 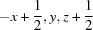.

**Table 2:** Hydrogen-bond geometry (Å, °)

*D*—H⋯*A*	*D*—H	H⋯*A*	*D*⋯*A*	*D*—H⋯*A*
O3—H31⋯O1^ii^	0.83 (5)	1.96 (5)	2.786 (3)	176 (5)
O3—H32⋯O2^iii^	0.94 (4)	1.75 (4)	2.672 (3)	167 (4)

Symmetry codes: (ii) 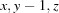; (iii) 
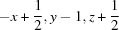
.

**Fig. 1.**
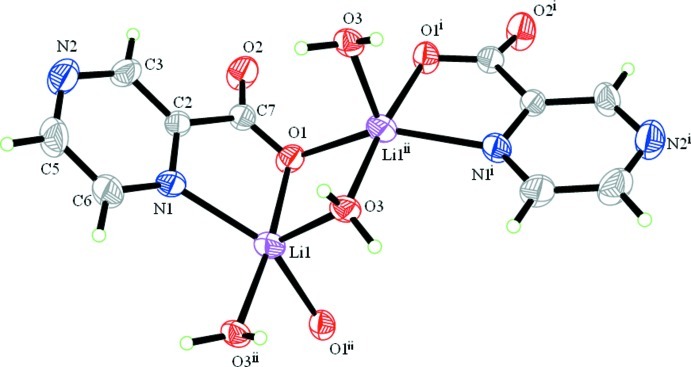
Two structural units of the title compound with atom labelling scheme and 50% probability displacement ellipsoids. Symmetry code: (i) -x + 1/2, y, z - 1/2; (ii) -x + 1/2, y, z + 1/2.

**Fig. 2.**
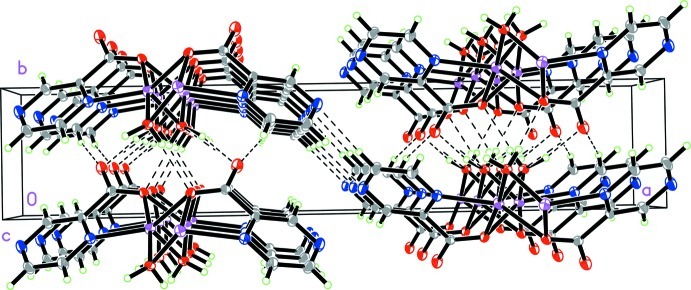
Packing diagram of the structure viewed along the c axis.

## supplementary crystallographic information

### Comment

The structure of the title complex is built of Li^I^ ions, each coordinated by ligand with *N1,O1* where O atom acts as bidentate and bridging to symmetry related Li1 and Li1^i^ ions, whereas the O2 atom remains chelating inactive. The metal ions are also bridged by coordinated water O3 atom forming a Li1—O1—Li1^i^—O3—Li1 connectivity with Li1—Li1^i^ distance of 3.052 (4) Å, (Fig.1). The observed bonding pathways –Li—O_carb_—Li- and –Li—O_aqua_—Li- give rise to molecular ribbon which propagates in the unit cell *c* direction (Fig. 2). The Li1 coordination polyhedron is distorted trigonal bipyramid (Fig. 1, Table 1) with an equatorial plane composed of O1, N1^i^ and O3^i^; the Li1 ion is 0.0405 (2) Å out of the plane, O1 and O3 atoms are at the axial positions. The pyrazine ring is planar with r.m.s. of 0.0019 (1) Å; the dihedral angle between the pyrazine and the carboxylato group (C7/O1/O2) is 12.3 (1)°. Hydrogen bonds are realised through coordinated aqua O3 and carboxylato O2 atoms (Table 2, Fig. 2). Weak C—H···N interactions of 3.518 (5) Å and 3.651 (5) Å are observed. The structures of Li^I^ complexes with diazine monocarboxylate ligands show a variety of polymeric patterns. The structure of a complex with 3-aminopyrazine-2-carboxylato ligand shows a catenated pattern (Starosta & Leciejewicz, 2010) while the structure of a complex with 5-methylpyrazine-2-carboxylato ligand is composed of molecular columns (Starosta & Leciejewicz, 2011*a*). Molecular layers were reported in the structure of a complex with pyrimidine-2-carboxylato and nitrato ligands (Starosta & Leciejewicz, 2011*d*) and in the structure of a complex with pyridazine-4-carboxylato ligand (Starosta & Leciejewicz, 2011*c*). On the other hand, the structure of a complex with pyridazine-3-carboxylato ligand is built of monomeric molecules (Starosta & Leciejewicz, 2011*b*).

### Experimental

50 mL of a solution containing 1 mmol of LiNO_3_ and an excess of pyrazine-2-carboxylic acid dihydrate to mantain pH *ca* 5.1 was boiled under reflux with stirring for 10 h, then left to crystallise at room temperature. After a couple of days single-crystal blocks of the title compound were detected among polycrystalline material. They were washed with methanol and dried in the air.

### Refinement

Water hydrogen atoms were located in a difference map and refined isotropically while H atoms attached to pyrazine-ring C atoms were positioned at calculated positions and were treated as riding on the parent atoms, with C—H=0.93 Å and *U*_iso_(H)=1.2*U*_eq_(C).

### Crystal data

**Table:**

[Li(C_5_H_3_N_2_O_2_)(H_2_O)]	*F*(000) = 304
*M**_r_* = 148.05	*D*_x_ = 1.491 Mg m^−^^3^
Orthorhombic, *P**c**a*2_1_	Mo *K*α radiation, λ = 0.71073 Å
Hall symbol: P 2c -2ac	Cell parameters from 25 reflections
*a* = 24.433 (5) Å	θ = 6–15°
*b* = 4.7861 (10) Å	µ = 0.12 mm^−^^1^
*c* = 5.6385 (11) Å	*T* = 293 K
*V* = 659.4 (2) Å^3^	Blocks, colourless
*Z* = 4	0.35 × 0.18 × 0.13 mm

### Data collection

**Table:**

Kuma KM-4 four-cricle diffractometer	813 reflections with *I* > 2σ(*I*)
Radiation source: fine-focus sealed tube	*R*_int_ = 0.078
Graphite monochromator	θ_max_ = 30.1°, θ_min_ = 1.7°
profile data from ω/2θ scans	*h* = −27→34
Absorption correction: analytical (*CrysAlis RED*; Oxford Diffraction, 2008)	*k* = 0→6
*T*_min_ = 0.972, *T*_max_ = 0.995	*l* = 0→7
1586 measured reflections	3 standard reflections every 200 reflections
1056 independent reflections	intensity decay: 4.4%

### Refinement

**Table:**

Refinement on *F*^2^	Primary atom site location: structure-invariant direct methods
Least-squares matrix: full	Secondary atom site location: difference Fourier map
*R*[*F*^2^ > 2σ(*F*^2^)] = 0.041	Hydrogen site location: inferred from neighbouring sites
*wR*(*F*^2^) = 0.116	H atoms treated by a mixture of independent and constrained refinement
*S* = 1.09	*w* = 1/[σ^2^(*F*_o_^2^) + (0.0244*P*)^2^ + 0.4211*P*] where *P* = (*F*_o_^2^ + 2*F*_c_^2^)/3
1056 reflections	(Δ/σ)_max_ < 0.001
108 parameters	Δρ_max_ = 0.31 e Å^−^^3^
1 restraint	Δρ_min_ = −0.30 e Å^−^^3^

### Special details

**Table:**

Geometry. All e.s.d.'s (except the e.s.d. in the dihedral angle between two l.s. planes) are estimated using the full covariance matrix. The cell e.s.d.'s are taken into account individually in the estimation of e.s.d.'s in distances, angles and torsion angles; correlations between e.s.d.'s in cell parameters are only used when they are defined by crystal symmetry. An approximate (isotropic) treatment of cell e.s.d.'s is used for estimating e.s.d.'s involving l.s. planes.
Refinement. Refinement of *F*^2^ against ALL reflections. The weighted *R*-factor *wR* and goodness of fit *S* are based on *F*^2^, conventional *R*-factors *R* are based on *F*, with *F* set to zero for negative *F*^2^. The threshold expression of *F*^2^ > σ(*F*^2^) is used only for calculating *R*-factors(gt) *etc*. and is not relevant to the choice of reflections for refinement. *R*-factors based on *F*^2^ are statistically about twice as large as those based on *F*, and *R*- factors based on ALL data will be even larger.

### Fractional atomic coordinates and isotropic or equivalent isotropic displacement parameters (Å^2^)

**Table:**

	*x*	*y*	*z*	*U*_iso_*/*U*_eq_	
O1	0.28627 (9)	1.1874 (4)	0.7415 (4)	0.0324 (4)	
O2	0.35150 (10)	1.3781 (5)	0.5168 (5)	0.0480 (7)	
N1	0.36091 (10)	0.8665 (5)	0.9601 (5)	0.0320 (5)	
C2	0.37639 (11)	1.0110 (5)	0.7680 (5)	0.0258 (5)	
C7	0.33500 (11)	1.2093 (5)	0.6658 (5)	0.0281 (5)	
C5	0.44910 (14)	0.6601 (7)	0.9486 (7)	0.0453 (8)	
H5	0.4735	0.5346	1.0167	0.054*	
N2	0.46487 (12)	0.8025 (6)	0.7586 (6)	0.0462 (7)	
C6	0.39802 (14)	0.6901 (7)	1.0492 (6)	0.0414 (7)	
H6	0.3892	0.5851	1.1825	0.050*	
C3	0.42814 (12)	0.9788 (7)	0.6705 (6)	0.0364 (6)	
H3	0.4375	1.0848	0.5383	0.044*	
Li1	0.2739 (2)	0.9324 (11)	1.0354 (10)	0.0338 (10)	
O3	0.22904 (9)	0.6809 (4)	0.8254 (4)	0.0282 (4)	
H31	0.247 (3)	0.538 (8)	0.796 (9)	0.051 (11)*	
H32	0.1986 (18)	0.599 (8)	0.901 (7)	0.046 (11)*	

### Atomic displacement parameters (Å^2^)

**Table:**

	*U*^11^	*U*^22^	*U*^33^	*U*^12^	*U*^13^	*U*^23^
O1	0.0283 (8)	0.0332 (9)	0.0358 (10)	0.0041 (8)	0.0052 (9)	0.0123 (10)
O2	0.0369 (11)	0.0567 (13)	0.0504 (15)	0.0051 (10)	0.0081 (11)	0.0333 (12)
N1	0.0324 (12)	0.0371 (11)	0.0265 (11)	0.0009 (10)	0.0033 (11)	0.0109 (10)
C2	0.0244 (10)	0.0276 (10)	0.0254 (11)	−0.0016 (10)	0.0010 (10)	0.0055 (10)
C7	0.0288 (11)	0.0282 (11)	0.0274 (12)	0.0006 (10)	0.0000 (11)	0.0069 (12)
C5	0.0361 (16)	0.0481 (17)	0.0517 (19)	0.0118 (14)	−0.0062 (17)	0.0153 (16)
N2	0.0333 (13)	0.0540 (16)	0.0514 (17)	0.0105 (12)	0.0064 (13)	0.0105 (16)
C6	0.0388 (16)	0.0493 (17)	0.0362 (15)	0.0036 (13)	−0.0019 (14)	0.0205 (15)
C3	0.0311 (13)	0.0433 (15)	0.0347 (14)	0.0014 (12)	0.0098 (13)	0.0093 (14)
Li1	0.036 (3)	0.040 (2)	0.026 (2)	−0.002 (2)	0.001 (2)	0.007 (2)
O3	0.0328 (9)	0.0287 (9)	0.0230 (8)	−0.0004 (8)	0.0014 (8)	0.0075 (9)

### Geometric parameters (Å, º)

**Table:**

O1—C7	1.269 (4)	N2—C3	1.328 (4)
Li1—O1	2.080 (6)	C6—H6	0.9300
O1—Li1^i^	2.237 (6)	C3—H3	0.9300
O2—C7	1.233 (4)	Li1—O3	2.013 (6)
N1—C6	1.337 (4)	Li1—O3^ii^	2.032 (5)
N1—C2	1.340 (4)	Li1—O1^ii^	2.237 (6)
Li1—N1	2.190 (6)	Li1—Li1^i^	3.052 (4)
C2—C3	1.387 (4)	Li1—Li1^ii^	3.052 (4)
C2—C7	1.502 (4)	O3—Li1^i^	2.032 (5)
C5—N2	1.327 (5)	O3—H31	0.83 (5)
C5—C6	1.379 (5)	O3—H32	0.94 (4)
C5—H5	0.9300		
			
C7—O1—Li1	116.9 (2)	O3—Li1—N1	109.2 (3)
C7—O1—Li1^i^	119.2 (2)	O3^ii^—Li1—N1	96.0 (2)
Li1—O1—Li1^i^	89.92 (19)	O1—Li1—N1	77.8 (2)
C6—N1—C2	116.0 (3)	O3—Li1—O1^ii^	105.9 (3)
C6—N1—Li1	132.6 (3)	O3^ii^—Li1—O1^ii^	83.2 (2)
C2—N1—Li1	110.9 (2)	O1—Li1—O1^ii^	100.9 (2)
N1—C2—C3	121.4 (3)	N1—Li1—O1^ii^	144.8 (3)
N1—C2—C7	116.5 (2)	O3—Li1—Li1^i^	41.25 (17)
C3—C2—C7	122.1 (2)	O3^ii^—Li1—Li1^i^	136.9 (2)
O2—C7—O1	126.1 (3)	O1—Li1—Li1^i^	47.12 (13)
O2—C7—C2	117.1 (3)	N1—Li1—Li1^i^	101.1 (2)
O1—C7—C2	116.8 (2)	O1^ii^—Li1—Li1^i^	103.2 (3)
N2—C5—C6	122.8 (3)	O3—Li1—Li1^ii^	109.5 (3)
N2—C5—H5	118.6	O3^ii^—Li1—Li1^ii^	40.79 (14)
C6—C5—H5	118.6	O1—Li1—Li1^ii^	142.33 (19)
C5—N2—C3	115.6 (3)	N1—Li1—Li1^ii^	123.4 (3)
N1—C6—C5	121.7 (3)	O1^ii^—Li1—Li1^ii^	42.96 (17)
N1—C6—H6	119.2	Li1^i^—Li1—Li1^ii^	135.0 (4)
C5—C6—H6	119.2	Li1—O3—Li1^i^	98.0 (2)
N2—C3—C2	122.5 (3)	Li1—O3—H31	109 (4)
N2—C3—H3	118.7	Li1^i^—O3—H31	110 (3)
C2—C3—H3	118.7	Li1—O3—H32	114 (3)
O3—Li1—O3^ii^	95.7 (2)	Li1^i^—O3—H32	126 (2)
O3—Li1—O1	87.8 (2)	H31—O3—H32	100 (4)
O3^ii^—Li1—O1	173.7 (3)		

Symmetry codes: (i) −*x*+1/2, *y*, *z*−1/2; (ii) −*x*+1/2, *y*, *z*+1/2.

### Hydrogen-bond geometry (Å, º)

**Table:**

*D*—H···*A*	*D*—H	H···*A*	*D*···*A*	*D*—H···*A*
O3—H31···O1^iii^	0.83 (5)	1.96 (5)	2.786 (3)	176 (5)
O3—H32···O2^iv^	0.94 (4)	1.75 (4)	2.672 (3)	167 (4)

Symmetry codes: (iii) *x*, *y*−1, *z*; (iv) −*x*+1/2, *y*−1, *z*+1/2.
